# Surgical treatment for breast cancer in a patient with erythropoietic protoporphyria and photosensitivity: a case report

**DOI:** 10.1186/s40792-020-01068-5

**Published:** 2021-01-05

**Authors:** Akiko Shimazaki, Takuma Hashimoto, Masaya Kai, Tetsuzo Nakayama, Mai Yamada, Karen Zaguirre, Kentaro Tokuda, Makoto Kubo, Ken Yamaura, Masafumi Nakamura

**Affiliations:** 1 Department of Surgery and Oncology, Graduate School of Medical Sciences, Kyushu University, 3-1-1 Maidashi, Higashi-ku, Fukuoka, 812-8582 Japan; 2 Department of Anesthesiology and Critical Care Medicine, Graduate School of Medical Sciences, Kyushu University, 3-1-1 Maidashi, Higashi-ku, Fukuoka, 812-8582 Japan; 3 Operating Room, Kyushu University Hospital, 3-1-1 Maidashi, Higashi-ku, Fukuoka, 812-8582 Japan; 4 Emergency and Critical Care Center, Kyushu University Hospital, 3-1-1 Maidashi, Higashi-ku, Fukuoka, 812-8582 Japan

**Keywords:** Erythropoietic protoporphyria (EPP), Photosensitivity, Breast cancer surgery

## Abstract

**Background:**

Erythropoietic protoporphyria (EPP) is a rare disorder of heme synthesis. Patients with EPP mainly show symptoms of photosensitivity, but approximately 20% of EPPs are associated with the liver-related complications. We report a case of breast cancer in a 48-year-old female patient with EPP in whom meticulous perioperative management was required in order to avoid complications resulting from this disease.

**Case presentation:**

The patient was diagnosed with EPP at the age of 33 and had a rich family history of the disease. For right breast cancer initially considered as TisN0M0 (Stage 0), the right mastectomy and sentinel lymph node biopsy were performed, while the final stage was pT1bN0M0, pStage I. In the perioperative period, we limited the drug use and monitored light wavelength measurements. Besides, we covered surgical lights, headlights, and laryngoscope’s light with a special polyimide film that filtered the wavelength of light causing dermal photosensitivity. After the surgery, any emerging complications were closely monitored.

**Conclusions:**

The surgery, internal medicine, anesthesiology, and operation departments undertook all possible measures through close cooperation to ensure a safe surgery for the patient with a rare condition.

## Background

Erythropoietic protoporphyria (EPP) is a genetic disorder caused by an abnormality in the ferrochelatase (*FECH*) gene involved in the heme synthetic pathway. This pattern of inheritance is said to be an incomplete autosomal dominant disorder, in which asymptomatic carriers are often found who do not develop the disease although they may have a clear familial mutation. The resulting decrease of FECH levels causes accumulation of protoporphyrin (PP) in the skin, plasma, erythrocytes, and liver that causes various disorders and symptoms [1, 2]. The main symptom is photosensitivity, but approximately 20% of EPPs are associated with the liver damage. However, less than 5% of EPPs can develop severe disease owing to cholestasis, and the liver transplantation remains the only life-saving treatment for patients with advanced liver failure [3, 4]. The incidence of EPP in the population is approximately 1:75,000 [5]. Of note, it is important to prevent phototoxic injury to skin and organs owing to the activation of PP by light. Here, we report that we performed breast cancer surgery on an EPP patient with photosensitivity. To the best of our knowledge, this is the first clinical report of breast cancer surgery in a patient with erythropoietic protoporphyria.

## Case presentation

The patient, a 48-year-old female with photosensitivity, was diagnosed with EPP at the age of 33. She had right breast pain and was diagnosed with breast cancer by the family doctor. She was referred to our department for surgical management. Her mother and two sisters, diagnosed as EPPs, died of liver failure.

There was a mass, 4 cm in size, in the lateral aspect of the right breast. Axillary lymph nodes were normal. She had no abdominal pain and no neurological symptoms.

Tumor markers (CA15-3 and CEA) were in the normal range. There was no elevation of hepatic enzymes and the liver function was preserved. The PP level was elevated to 2924 μg/dL.

**Fig. 1:**
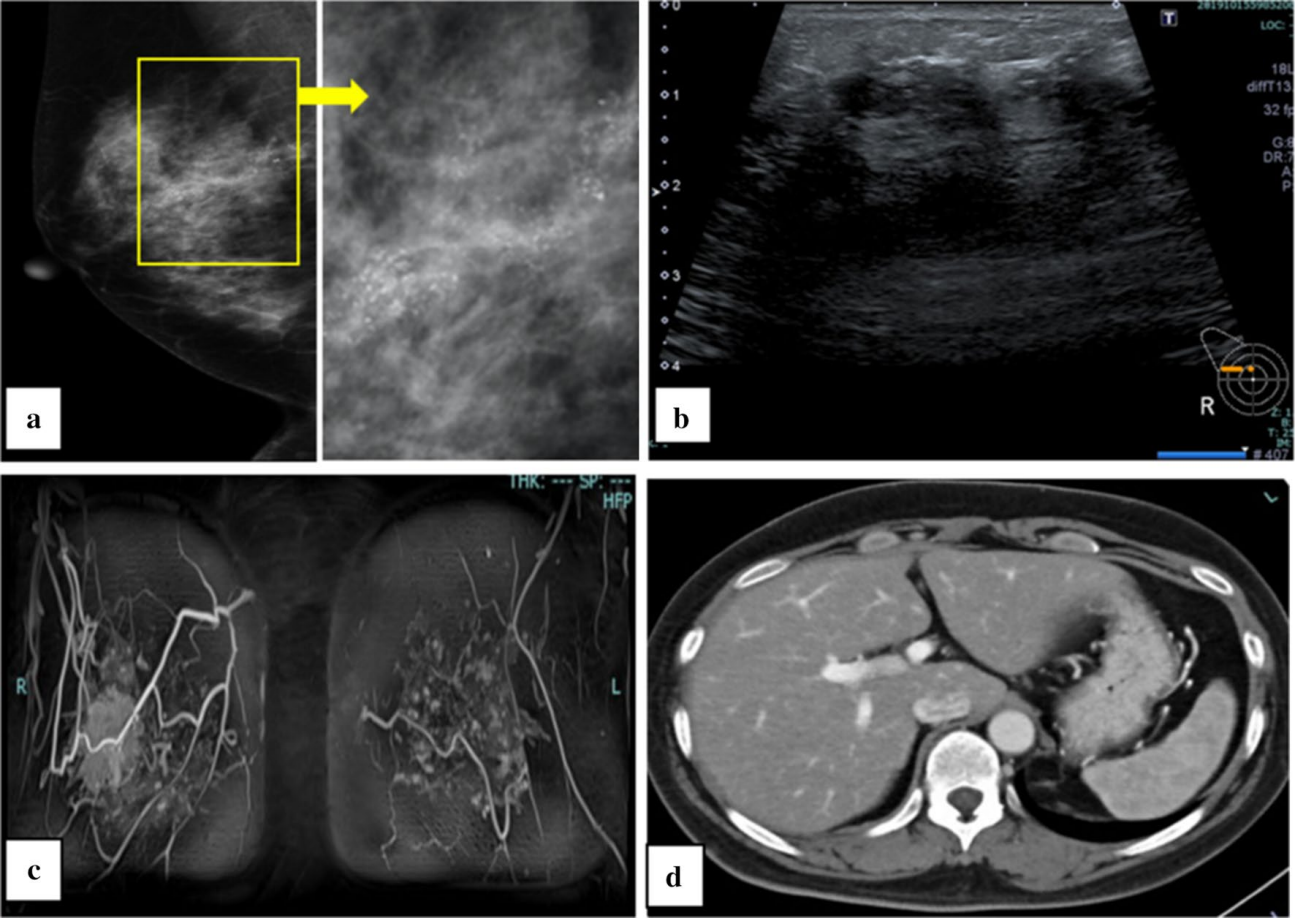
Imaging findings. **a** Mammogram shows segmental polymorphic calcifications (yellow square) in the right ML-UM (BI-RADS 5). **b** Ultrasound revealed an axial 45 mm hypoechoic mass with internal coarse calcifications in the right breast (BI-RADS 5). **c** Contrast magnetic resonance imaging showed wide-range non-mass enhancement in the upper-outer quadrant of the right breast. **d** Contrast computed tomography showed that there was no distant metastasis and liver morphology was maintained and no gallstones were noted

Mammogram showed segmental polymorphic calcifications in the right breast (BI-RADS 5) (Fig. 1a). Ultrasound revealed an axial 45 mm hypoechoic mass with internal coarse calcifications in the right breast (BI-RADS 5) (Fig. 1b). On contrast magnetic resonance imaging, there was wide-range non-mass enhancement in the upper-outer quadrant of the right breast (Fig. 1c). Distant metastasis was not observed and liver morphology was maintained and no gallstones were noted on contrast computed tomography (Fig. 1d). An ultrasound-guided core-needle biopsy showed ductal carcinoma in situ. Therefore, for the right breast cancer (TisN0M0 Stage 0), right mastectomy and sentinel lymph node biopsy were planned.

The following perioperative measures were undertaken, such as re-evaluation of EPP, measures against photosensitivity, and confirmation of drugs used.

**Fig. 2:**
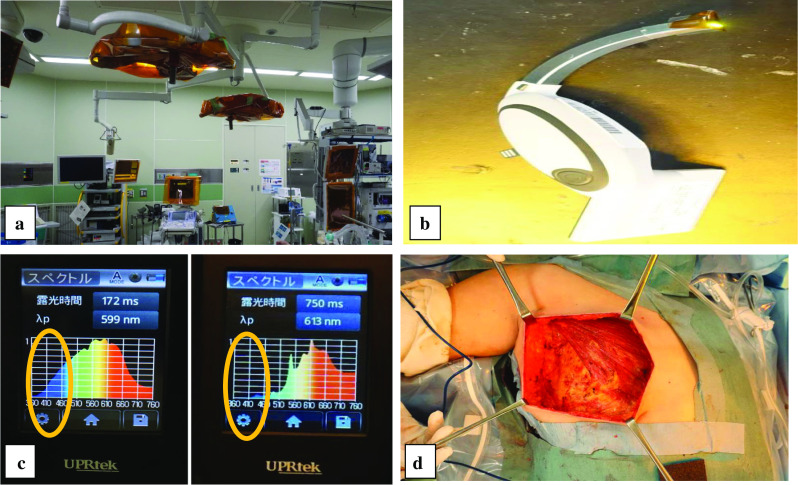
Modifications to the operating room. **a** In the operating room, several emitters are covered with the special film. The light intensity of the ceiling light did not change the 400 nm region of the light wavelength measured on the surgical table, so for the convenience of the surgery, the ceiling light was not filtered. **b** The laryngoscope’s light is also covered with the film. **c** We measured the lights at the operation table. We confirmed that the film completely blocked light with a wavelength of 400 nm. (Left) without the film, (right) with film attached (yellow circle). **d** We could safely manage the operation in a sufficiently bright environment

**Fig. 3:**
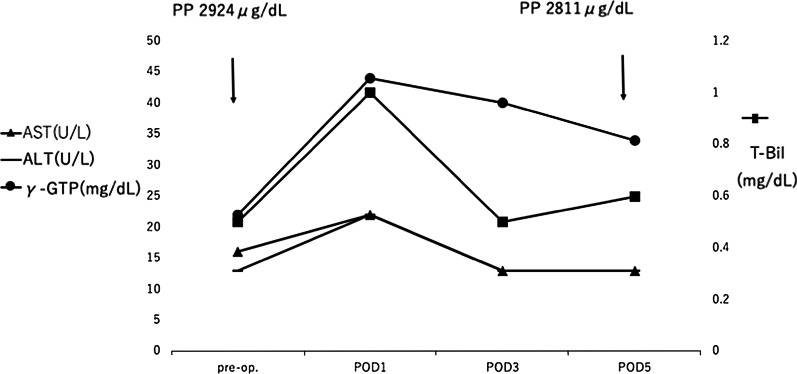
Lab parameters showing stable liver function. The postoperative course was uneventful, and the level of PP and liver function were stable

1. Preoperative preparation: by consulting with hepatologist, it was confirmed that the current liver function was maintained and that the PP level was stable.
2. Measures against light wavelength: photosensitive symptoms of EPP have been reported to be triggered by wavelengths of light around 400 nm [6–9]. The day before the surgery, to simulate the procedure, we covered surgical lights, headlights, and anesthesia monitors in the operating room with the special film (Kapton^®^ polyimide film, DU PONT-TORAY CO., LTD.) (Fig. 2a). The laryngoscope’s light also contained the 400-nm-region wave that was also blocked by attaching the film (Fig. 2b). We planned to intubate the patient using a videolaryngoscope (McGRATH^®^) because it was predicted that the view would be dark rendering the intubation difficult. Moreover, we confirmed that the film completely blocked light with a wavelength of 400 nm by a spectroradiometer (MK-350, United Power Research Technology Corp, Miaoli County, Taiwan) (Fig. 2c). Although the operative field was dim yellow-to-orange because of the influence of the film (Fig. 2d), there was no difficulty in identifying the tissues, and the surgery was possible as usual.
3. Induction and maintenance of anesthesia: we chose total intravenous anesthesia (TIVA) with target-controlled infusion (TCI). Standard monitors were placed, including blood pressure cuff, pulse oximetry, and electrocardiography. Anesthesia was induced by intravenous administration of fentanyl, propofol, and rocuronium, and maintained with propofol (TCI of 2.5 µg/mL), 0.2 µg/mg/min of remifentanil, and a 50% oxygen–air mixture. Intubation was performed with the film-covered video laryngoscope. To manage postoperative pain, acetaminophen was administrated. The mastectomy was completed with an operating time of 1 h 48 min and total blood loss of 30 g. The patient’s urine volume was 150 ml. The endotracheal tube was extubated without difficulty, and patient reported no complaints of pain or nausea. No cutaneous or oral symptoms of photosensitivity were observed.
4. Sentinel lymph node biopsy: in our hospital, radio isotope (RI) method with phytate colloid, and dye method with indigo carmine are usually used together. Since the risk of indigo carmine was unknown for EPP cases, the RI method alone was used.
5. Postoperative management: to manage postoperative pain, acetaminophen was administered before the end of the surgery. The postoperative course was uneventful, and she was discharged 8 days after surgery without causing porphyria symptoms, such as liver damage (Fig. 3).

Final pathological diagnosis was invasive ductal carcinoma with a widespread DCIS. The maximum invasion and extension diameters were 10 mm and 100 mm, respectively. Biomarker analysis using immunohistochemistry showed estrogen receptor (ER) negativity, progesterone receptor (PR) negativity, and human epidermal growth factor receptor 2 (HER2) positivity. There was no vascular invasion and the nuclear grade was 3 (nuclear atypical: 3 points, mitotic figures: 2 points) [10], and the MIB-1 was positive in 30% tumor cells. No lymph node metastasis was observed and the final stage was pT1bN0M0, pStage I.

For postoperative treatment, chemotherapy and molecular-targeted drugs were examined according to the guidelines [11]. But for patients with EPP, the risk of chemotherapy and molecular-targeted drugs remain unknown, and there are many contraindicated drugs among the supporting drugs (antibacterial drugs and antiemetics). Moreover, she was a patient with early breast cancer (pT1b), so it was thought that the acute attack of porphyria would have had a greater impact on the prognosis than the risk of recurrence. After consulting with the patient, we decided to follow-up without treatment.

## Discussion

The porphyrias are genetic disorders, each resulting from the deficiency of the FECH enzyme in the heme biosynthetic pathway. These are eight genetically distinct metabolic disorders that can be classified as “acute hepatic”, “hepatic cutaneous”, and “erythropoietic cutaneous” diseases [12].

EPP is classified as the erythropoietic-cutaneous type, and characterized by photosensitivity appearing since infancy [12, 13]. However, photosensitivity occurs in patients with EPP when they receive light in the wavelength around 400 nm. The general operating room light is in the wavelength range of 300–750 nm and includes wavelengths in that region [7]. During the surgery, in addition to the skin, light exposure to various other organs should also be considered. There is a report of a patient with EPP who suffered perioperative third-degree skin burn and multiple intestinal perforations after liver transplantation surgery [14, 15]. On the other hand, it was also reported that EPP patients were operated on without the ray protection without encountering any problems [16, 17]. Since it is clear that the risk of complications can be sufficiently reduced by using the filter, we decided to perform the surgery using the film. In the absence of the film, the ceiling lights, surgical lights, and monitor contained wavelengths that peaked around 400 nm. The same was also true of the videolaryngoscope’s light, although it was not measured in other reports. The measurements were repeated with the film covered and it was confirmed that the light rays around that wavelength were blocked.

In the acute-hepatic type of porphyria, use of certain medications should be avoided because the symptoms are exacerbated by their use [18]. However, it is not necessary for the patients with EPP to avoid certain medications like the patient with acute-hepatic type of porphyria. Yet, there have been no reports of patients with EPP that used such drugs without problems, and the patient herself practiced drug restrictions for precaution. Therefore, in this case, the medication choice was based on acute intermittent porphyria (AIP), which causes an acute attack.

In this case, there were no major problems such as photosensitivity in daily life and liver dysfunction. However, acute exacerbations can be fatal, and the patient’s sisters are also undergoing a rapid course of the disease. Therefore, we carefully evaluated the following.

1. Re-evaluation of EPP by specialist: in general, if the PPs were low, the condition was considered to be stable. In this case, no marked changes of PPs, and no exacerbation of EPP were observed during the perioperative period.
2. Adjustment of hospital environment: the photosensitivity was not remarkable, so it was not necessary to manage the private room or the dark room as recommended in severe cases. There was no problem with avoiding the windows and curtain shading.
3. Medication confirmation: there are a wide variety of drugs that cause acute attacks. During the perioperative period, restricted use of many drugs such as antibiotics, analgesics, and anesthetics is considered. A strict medication confirmation was performed from introduction to pain management.
4. Devices used in surgical procedure: we performed standard breast cancer surgery. The effect of the indigo carmine dye on sentinel lymph node biopsy was unclear, therefore only the RI method was performed. Surgical lights and headlights contain a wavelength of 410 nm, which can cause photosensitivity, but it was possible to remove that wavelength by covering with the film. There was no significant effect on the surgical procedure.
5. Adjudication of adjuvant therapy: compared with benign diseases and other carcinomas, breast cancer treatment is characterized by a combination of drug therapy and radiation therapy in addition to surgery. Anticancer agents, molecular-targeting agents and hormonal agents are recommended depending on the risk of recurrence and subtype. However, there is no sufficient information on the safety of these agents in EPP. Furthermore, since a wide variety of supportive drugs such as antibiotics, antipyretic analgesics, antiemetics, antihistamines, and steroids would have been needed, and there was the concern regarding the adverse effects of these drugs on EPP. Radiation therapy is recommended for breast-conserving surgery and highly positive lymph node metastases, but there is no evidence of its safety in EPP. These adjuvant therapies are mainly aimed at suppressing the recurrence of breast cancer, but in this case, we considered that the indication of adjuvant therapy should be evaluated by balancing the risk of recurrence with that of exacerbation of EPP. This case was HER2 type, and thus anticancer drug plus trastuzumab was recommended. However, it was pT1bN0 and the recurrence risk was low even without treatment (5-year disease-free survival (DFS) of 90% [19], therefore we decided not to initiate adjuvant therapy.

In addition, in all the processes, it was possible to carry out examinations and treatments safely and reliably by cooperation from surgeons, physicians, anesthesiology, and operating departments.

## Conclusions

We experienced a rare case of breast cancer with EPP and photosensitivity who safely underwent breast surgery that was achieved through several special measures and close cooperation of different clinical departments.

## Data Availability

Please contact the corresponding author for data requests.

## Abbreviations

EPP: Erythropoietic protoporphyria
FECH: Ferrochelatase
PP: Protoporphyrin
RI: Radio isotope
NSAIDs: Non-steroidal anti-inflammatory drugs
ER: Estrogen receptor
PR: Progesterone receptor
HER2: Human epidermal growth factor receptor 2
AIP: Acute intermittent porphyria
DFS: Disease-free survival

## References

[1] Lecha M, Puy H, Deybach JC (2009). Erythropoietic protoporphyria. Orphanet J Rare Dis.

[2] Pagano MB, Hobbs W, Linenberger M, Delaney M (2012). Plasma and red cell exchange transfusions for erythropoietic protoporphyria: a case report and review of the literature. J Clin Apher.

[3] Kondo M, Yano Y, Shirataka M, Urata G, Sassa S (2004). Porphyrias in Japan: compilation of all cases reported through 2002. Int J Hematol.

[4] McGuire BM, Bonkovsky HL, Carithers RL, Chung RT, Goldstein LI, Lake JR (2005). Liver transplantation for erythropoietic protoporphyria liver disease. Liver Transpl.

[5] Park PJ, Hwang S, Choi YI, Yu YD, Park GC, Jung SW (2012). Liver transplantation for acute-on-chronic liver failure from erythropoietic protoporphyria. Clin Mol Hepatol.

[6] Magnus IA, Jarrett A, Prankerd TA, Rimington C (1961). Erythropoietic protoporphyria: a new porphyria syndrome with solar urticaria due to protoporphyrinaemia. Lancet.

[7] Asokumar B, Kierney C, James TW, Amato J, Tumanmd KJ (1999). Anaesthetic management of a patient with erythropoietic protoporphyria for ventricular septal defect closure. Paediatr Anaesth.

[8] Hanaki T, Noda T, Eguchi H, Iwagami Y, Akita H, Asaoka T (2020). Successful liver transplantation for liver failure with erythropoietic protoporphyria by covering the operating theater lights with polyimide film: a case report. Transplant Proc.

[9] Wahlin S, Srikanthan N, Hamre B, Harper P, Brun A (2008). Protection from phototoxic injury during surgery and endoscopy in erythropoietic protoporphyria. Liver Transpl.

[10] http://jbcs.gr.jp/guidline/2018/index/byouri/ (**in Japanese**). Accessed 7 Dec 2020.

[11] http://jbcs.gr.jp/guidline/2018/index/yakubutu/ (**in Japanese**). Accessed 7 Dec 2020.

[12] Balwani M, Desnick RJ (2012). The porphyrias: advances in diagnosis and treatment. Blood.

[13] Bissell DM, Anderson KE, Bonkovsky HL (2017). Porphyria. N Engl J Med.

[14] Herbert A, Corbin D, Williams A, Thompson D, Buckels J, Elias E (1991). Erythropoietic protoporphyria: unusual skin and neurological problems after liver transplantation. Gastroenterology.

[15] Seth AK, Badminton MN, Mirza D, Russell S, Elias E (2007). Liver transplantation for porphyria: who, when, and how?. Liver Transpl.

[16] Akinci E, Erentug V, Bozbuga N, Polat A, Mansuroglu D, Yakut C (2005). Combined valve and coronary surgery in a patient with erythropoietic protoporphyria. J Card Surg.

[17] Roe T, Bailey IS (2010). Laparoscopic cholecystectomy in a patient with erythropoietic protoporphyria. J Surg Case Rep.

[18] Stölzel U, Doss MO, Schuppan D (2019). Clinical guide and update on porphyrias. Gastroenterology.

[19] Kubo M, Kawai M, Kumamaru H, Miyata H, Tamura K, Yoshida M (2019). A population-based recurrence risk management study of patients with pT1 node-negative HER2+ breast cancer: a National Clinical Database study. Breast Cancer Res Treat.

